# Introducing the Metaphorical Anchor Model: A Mixed-Methods Study of Cognitive Metaphor Use in Palliative Care

**DOI:** 10.3390/bs16071148

**Published:** 2026-07-08

**Authors:** Margherita Dahò

**Affiliations:** Department of Psychology, Educational Science and Human Movement, University of Palermo, Viale delle Scienze, Ed. 15, 90146 Palermo, Italy; margherita.daho@unipa.it

**Keywords:** comfort care, communication, death, general psychology, healthcare, language, metaphor, palliative care, perinatal hospice, symbols

## Abstract

This study introduces and provides an initial validation of the Metaphorical Anchor Model, a framework in which metaphors function as cognitive tools that transform affective overload into structured professional meaning in the context of death and end-of-life care. A mixed-methods design was used to test whether professional background moderates the anchoring process. Study 1 involved a qualitative analysis of 61 metaphorical expressions produced by 14 multidisciplinary professionals, using thematic analysis within a discourse-based interpretative framework grounded in cognitive linguistics. Study 2 provided quantitative validation through Fisher’s Exact Test to examine whether professional training influences the selection of metaphor types. Qualitative findings identified six cognitive domains grouped into two macro-clusters: Symbolic & Relational Anchors (symbolic, affective, relational) and Functional & Cognitive Analogies (embodied, spatial, cognitive). Quantitative results supported the model, showing professional role as a significant moderator (*p* = 0.008): science-based professionals predominantly used Functional & Cognitive Analogies (54.3%), whereas humanistic-psychosocial professionals relied mainly on Symbolic & Relational Metaphor Anchors (80.8%). These findings suggest that metaphor use offers insight into how professional roles shape representations of death and care and may represent a relevant resource for emotional processing. Beyond its theoretical contribution, the model has potential implications for clinical communication training, supporting healthcare professionals in managing emotionally demanding interactions and improving reflective practice in end-of-life care.

## 1. Introduction

Metaphors and analogies are figures of speech that use figurative language to represent one thing as another for rhetorical and meaning-making purposes (Guo, 2023; Hu, 2023). As noted by Lakoff and Johnson (1980), metaphors allow individuals to express and organize personal meanings, enabling them to “comprehend partially what cannot be comprehended totally” (p. 193). For this reason, metaphorical language is widely used not only in the literature but also in psychotherapeutic and clinical contexts, where it provides a means to articulate complex, emotionally charged, or otherwise ineffable experiences.

From a cognitive perspective, metaphors are not merely expressive devices but function as mental models through which individuals conceptualize abstract and emotionally complex phenomena (Fan, 2018; Holyoak & Stamenković, 2018; Tanaka et al., 2025). According to theories of embodied cognition, metaphors act as bridges between sensory experience and abstract thought, transforming perceptual inputs, such as images of space, movement, or light, into structured meanings that can be remembered, narrated, and shared (Beknazarova et al., 2021; Fan, 2018; Gibbs, 2005, 2011; Hu, 2023; Maslen, 2016). In this sense, metaphors integrate perception, language, and autobiographical memory, allowing individuals to map concrete experiential domains onto abstract emotional experiences and to construct coherent personal and professional meanings (Guo, 2023; Holyoak & Stamenković, 2018).

This integrative function of metaphors is consistent with Bucci’s (2021) multiple code theory, which posits that human cognition operates across verbal, symbolic, and sub-symbolic systems. Sub-symbolic emotional experiences are often more readily accessed through images than through literal language, making metaphors, analogies, and symbols particularly effective vehicles for capturing implicit meanings. Empirical studies suggest that the use of images and metaphorical representations in clinical contexts can facilitate the recall and reorganization of emotional memories (Fetterman et al., 2016; Yu et al., 2025), support grief processing and resilience following loss (Camsuzou, 2021; Sheridan & Van Lith, 2025), and promote adaptive reconstruction of experiences that may otherwise remain defensively dissociated from consciousness (Ecker et al., 2024; Graves, 2009). In this sense, metaphorical imagery may also function as a mnemonic and regulatory device, supporting emotional integration and reflective meaning-making.

Despite death being a universal human experience, public and professional discourse surrounding death and dying remains culturally sensitive and, in many societies, taboo. Conceptualizations of death are shaped by cultural, social, cognitive, and spiritual frameworks, which influence both death-related anxiety and how individuals perceive, remember, and assign meaning to dying (De Souza et al., 2024; Söylev et al., 2025). Within this context, metaphors may serve as cognitive–emotional mediators, allowing individuals to organize perceptions of death and integrate them into broader mental and experiential models.

Several studies have examined the use of metaphors in end-of-life and palliative care settings (Southall, 2013). Back et al. (2023), for example, identified “deep metaphors” associated with palliative care through interviews with patients and caregivers, demonstrating how metaphorical language shapes understanding and communication around serious illness. Similarly, Guité-Verret et al. (2021) showed that bereaved family caregivers rely on metaphors such as “cut-offs,” “obstructions,” and “shockwaves” to articulate the disruptive nature of grief. In perinatal contexts, Dahò (2020) found that American and Italian parents receiving personalized comfort care used metaphors to express emotions, make sense of loss, and communicate with healthcare providers. More recently, Gregory et al. (2025) examined the pervasive “journey” metaphor in conceptualizations of death, illustrating how metaphor structures cultural, psychological, and medical understandings of mortality. Collectively, these studies underscore the central role of metaphors in communication, emotional processing, and meaning-making in healthcare (Southall, 2013; Spall et al., 2001; Tay, 2016).

While existing research has primarily focused on patients’ and families’ metaphorical representations, less attention has been paid to how healthcare professionals themselves conceptualize death and caregiving through metaphor, particularly in perinatal hospice settings. From a cognitive and phenomenological standpoint, analyzing metaphorical language offers insight into how language, perception, and memory interact to shape professionals’ mental representations of end-of-life care (Mahon & Kemmerer, 2020; Pellerin et al., 2025). The present study builds on previous work exploring the emotional experiences and representations of healthcare professionals in perinatal hospice contexts (Dahò, 2021), extending this line of inquiry by focusing explicitly on metaphorical meaning-making. In perinatal hospice and neonatal intensive care contexts, healthcare professionals are routinely exposed to emotionally intense clinical experiences, including fetal or neonatal loss, parental grief, and ethically complex decision-making (Dahò, 2021). Although these experiences are not directly measured in the present study, their presence is well documented in the literature (e.g., Ravaldi et al., 2023) and is reflected in professionals’ spontaneous linguistic productions.

Before outlining the study aims, it is important to briefly describe the context of perinatal hospice care, which remains relatively unfamiliar in many clinical and academic settings, compared to adult palliative care units (Marc-Aurele, 2020). Perinatal hospice programs support families who continue a pregnancy following a prenatal diagnosis of a life-limiting condition or in cases of extremely preterm birth. Although such programs vary in structure, they share core principles, including comfort-focused care, multidisciplinary teamwork, ongoing compassionate communication, and individualized Prenatal Birthing Plans that reflect family values (Parravicini et al., 2018; Wool & Parravicini, 2020). Psychosocial support, facilitation of parent–infant bonding, and the creation of meaningful memories, such as photographs, narrative keepsakes, or memory boxes, are central components of care (Côté-Arsenault & Denney-Koelsch, 2018; Dahò, 2024, 2026; Porch et al., 2023; Thornton et al., 2021). Within this emotionally complex framework, metaphorical and image-based language may play a crucial role in supporting communication, emotional regulation, and meaning integration for both families and care providers.

### Purpose of the Study

The present research aimed to investigate how perinatal hospice care providers use metaphors as cognitive linguistic tools to construct, organize, and communicate their representations of death and end-of-life care. To achieve a comprehensive, theoretically grounded understanding of this phenomenon, the research was structured as a two-study mixed-methods design that integrated qualitative model development with quantitative validation. Given the exploratory nature of metaphor use in this context, the research questions were primarily designed to guide inductive qualitative inquiry in Study 1, whereas Study 2 was structured to test specific hypotheses derived from the qualitative model.

*Study 1: Qualitative Exploration and Model Development.* The first study aimed to identify recurring metaphorical schemas and symbolic themes used by perinatal hospice professionals in describing death, care, and emotional experience. This preliminary phase sought to examine how metaphors function as a cognitive bridge between raw, sub-symbolic emotional experiences and professional meaning-making within high-stress care contexts. Based on these qualitative findings, Study 1 aimed to develop a conceptual framework, the Metaphorical Anchor Model, that describes the cognitive and emotional functions of metaphors in hospice care.

- Q1: What are the predominant metaphorical schemas used by perinatal hospice providers to conceptualize death and comfort-focused care?
- Q2: How do these metaphors facilitate the transition from affective overload to cognitive integration and professional meaning-making?

*Study 2: Quantitative Validation and Comparative Analysis*. The second study aimed to empirically test key assumptions of the Metaphorical Anchor Model using a comparative quantitative design. Specifically, this phase examined whether professional background influences both the selection and functional role of metaphorical “anchors” in processing emotionally intense clinical experiences.

- Q3: Is there a significant association between providers’ professional background (science-based vs. humanistic/psychosocial) and the type of metaphorical anchors employed?

**Hypothesis** **1.** *Providers with a humanistic/psychosocial background will rely more heavily on symbolic and spatial anchors, while science-based providers will show a preference for functional and biological analogies. (The formulation of a single hypothesis reflects the confirmatory and theory-testing nature of Study 2, which was explicitly designed to examine one primary prediction derived from Study 1).*

## 2. Study 1: Materials and Methods

### 2.1. Participants

Fourteen care providers working in three public perinatal hospice programs in the United States, in New York City (State of New York) and Boston (State of Massachusetts), participated in the study. The sample included three physicians, four nurses, three social workers, two psychologists, and two Child Life specialists, representing the entire multidisciplinary team involved in neonatal palliative care. All participants were female, aged 27 to 62 years (Mean age = 47.2 years), with an average of 14 years of professional experience in neonatal or palliative care settings. Participants were recruited voluntarily and provided written informed consent before participation. The study was conducted in accordance with the principles of the Declaration of Helsinki (Bierer, 2025). As no sensitive or personally identifiable information was collected, all data were fully anonymized, and no intrusive or clinically sensitive questions were included, formal ethical approval from the local institutional review board was not required in accordance with applicable institutional and regulatory guidelines. Data processing complied with the General Data Protection Regulation (GDPR, Regulation (EU) 2016/679), ensuring participants’ privacy and confidentiality.

### 2.2. Procedure

Interviews were conducted individually in a private room within each hospice unit to ensure confidentiality and comfort, or online when in-person meetings were not possible. Each session began with a few general socio-demographic questions (age, role, and years of experience) and a brief description of the participant’s professional role within the hospice. This introductory phase served both to establish an initial connection and to help the interviewer better understand the units’ organizational and interpersonal dynamics. The interview then focused on two main topics: death and the perinatal hospice experience (commonly referred to by staff as “Comfort Care”). Participants were invited to reflect on their perceptions and emotional experiences of these themes and to choose or create an image, symbol, or metaphor that represents their personal view. This approach allowed participants to express both the linguistic and perceptual dimensions of their internal representations. Each interview lasted approximately 15 min, was audio recorded with the participant’s permission, and was transcribed verbatim for analysis. Interviews were conducted in English by the study’s author, a licensed psychologist and Ph.D. expert in qualitative research.

### 2.3. Interview Guide

The interview guide was developed based on theories from cognitive linguistics and embodied cognition (Fan, 2018; Guo, 2023; Holyoak & Stamenković, 2018). Questions encouraged participants to move from literal descriptions toward symbolic and metaphorical thinking, facilitating access to embodied and perceptual schemas underlying their professional experiences.

Example questions included: “If you were to describe death as an image, symbol, or metaphor, what would it be and why?”; “Which sensations, images, or concepts come to mind when you think about Comfort Care? Try to describe the unit with an analogy, symbol, or metaphor.”

This approach functioned as both a reflective and elicitative tool, allowing metaphorical expressions to emerge as indicators of cognitive and emotional processing.

### 2.4. Data Analysis

First, data were analyzed using Thematic Analysis following the six-step framework proposed by Braun and Clarke (2021). In this study, data referred specifically to metaphorical expressions, analogies, and symbolic images produced by participants when describing death and perinatal hospice care (Section 3.1 and Section 3.2). The analytic process included the following steps:

1. Familiarization with the data through repeated reading of transcripts, with particular attention to metaphorical language, symbolic images, and figurative expressions;
2. Generation of initial codes by identifying explicit metaphors, analogies, and symbolic references, as well as salient linguistic expressions conveying implicit figurative meaning;
3. Collation of codes into potential themes, grouping metaphorical expressions that reflected similar conceptual structures or shared underlying source domains;
4. Review and refinement of themes, ensuring internal consistency and conceptual coherence across metaphorical patterns;
5. Definition and naming of themes, focusing on the cognitive and emotional meanings expressed by participants rather than on surface linguistic features;

After this thematic structure had been established, a second interpretive step examined metaphors as indicators of underlying conceptual organization. The present study adopts an applied, discourse-based approach to metaphor analysis, drawing primarily on the framework outlined by Cameron and Maslen (2010). Unlike Conceptual Metaphor Theory (CMT; Lakoff & Johnson, 1980; Gibbs, 2011), which primarily identifies underlying conceptual metaphors through researcher-generated example sentences, Cameron and Maslen’s approach examines metaphors as they naturally emerge in discourse.

The metaphors are treated not as fixed conceptual structures but as dynamic linguistic phenomena that unfold in interaction and reflect the interplay of cognitive, embodied, affective, and socio-cultural processes (Cameron & Maslen, 2010; Maslen, 2016). Depending on the analytical focus, metaphors can thus be examined at multiple levels: linguistic, embodied, cognitive, affective, relational, or symbolic (Cameron & Maslen, 2010; Maslen, 2016). This discourse-based approach was preferred not because it is inherently superior to CMT, but because it is better suited to exploring how care providers cognitively and emotionally structure their experiences through metaphorical language.

Third, metaphors were identified within interview discourse and interpreted in terms of recurring cognitive domains and experiential schemas, including spatial (e.g., movement, boundaries, passage), embodied (e.g., holding, weight, warmth), relational (e.g., family, circle, connection), affective (e.g., peace, fear, relief), and symbolic or spiritual dimensions (Section 3.3). For example, outside the clinical context, expressions like “*learning is a journey*” or “*being stuck in a problem*” map abstract cognitive processes onto spatial or embodied experiences, while organizational metaphors such as “*building a foundation*” or “*navigating change*” structure complex activities through familiar experiential domains. This inductive grouping of metaphors by source domain enabled the analysis to capture both the systematicity and variability of metaphor use, supporting a cognitively informed interpretation grounded in real discourse. In this regard, a quantitative overview illustrates how care providers systematically map the abstract and threatening experience of death and palliative care onto more concrete cognitive frameworks.

Notably, to enhance credibility and reduce interpretative bias, analytic decisions regarding coding, theme development, and conceptual interpretation were discussed with two supervisors with complementary expertise in qualitative research. Divergent interpretations were critically examined and resolved through consensus, supporting the trustworthiness and transparency of the analytic process.

## 3. Study 1: Results

### 3.1. Results Related to the Death Topic

Forty-one statements on death and dying have been selected from interview transcripts and classified into 4 clusters: Peace, Natural event, Spirituality, and Unknown. The metaphors, themes, or considerations generated by the interviews are shown in Table 1.

#### 3.1.1. Peace (13 Statements)

The first theme, labeled Peace, comprises 13 statements and reflects the physical and emotional perception of death. Participants described death as quiet, without discomfort, peaceful, and free from suffering. From a cognitive standpoint, these metaphors suggest a mental process of emotional regulation and conceptual mapping, in which death is represented as a state of liberation from pain. For example, expressions such as “*there is a beautiful grace in a peaceful death*” or “*death is the end of pain*” indicate that care providers organize their perceptions of death into a conceptual schema of serenity and relief, allowing them to manage the otherwise threatening concept of mortality cognitively. This theme primarily draws on affective and embodied schemas (emotions of peace and the absence of pain; bodily relaxation).

#### 3.1.2. Natural Event (6 Statements)

The theme Natural event, composed of six statements, depicts death as a biological and inevitable process. Death is described as the “*cessation of the body’s functions*,” the “*completion of a journey*,” and “*part of everyone’s life*.” These metaphors reveal a conceptual mapping that anchors death in the familiar schema of natural life cycles. This framing reflects acceptance and resignation, indicating that professionals cognitively normalize death as a predictable, natural event. The theme relies mainly on embodied and temporal/spatial schemas (body functions, life progression).

#### 3.1.3. Spirituality (17 Statements)

The most frequent theme, Spirituality, includes 17 statements and represents a spiritual, transcendent conception of death. Metaphors such as “*death is the door to another part of life*” or “*death is going to a happy place*” reveal how participants map perceptual and experiential domains (light, journey, presence) onto abstract, spiritual concepts. Through these mappings, death is conceptualized not as an end but as a transformation and continuation. References to religious beliefs or spiritual figures indicate the integration of symbolic, affective, and relational schemas.

#### 3.1.4. Unknown (5 Statements)

The final theme, Unknown, comprises five statements that reflect the psychological and cognitive uncertainty surrounding death. Death is described as a “*big question mark*,” evoking fear due to its unpredictability and finality. These metaphors reveal gaps in conceptual mapping: unlike themes such as Spirituality, here care providers lack familiar perceptual or embodied schemas onto which they can project the abstract idea of death. The resulting cognitive tension illustrates the difficulty of organizing and integrating experiences of mortality when existing mental models are insufficient. This theme highlights the limits of metaphorical and cognitive strategies in making sense of the unknown, showing how uncertainty and unpredictability shape emotional responses and conceptual understanding. 

### 3.2. Results Related to Comfort Care

Twenty statements related to Comfort Care management were selected from the interview transcripts and classified into three categories: Circle/Union, Instrument, and House. The metaphors, themes, and considerations generated by the interviews are summarized in Table 2.

#### 3.2.1. Circle/Union (11 Statements)

The first theme comprises eleven statements and reflects the perception of Comfort Care as a relational and interactive practice. Metaphors such as “*the circle symbolizes the family unit and the medical team*” or comparing Comfort Care to a hug illustrate how participants cognitively map social and spatial schemas onto professional care practices. The circle metaphor embodies notions of containment, cohesion, and mutual support, highlighting how care providers mentally organize the interactions between family and medical staff. Statements such as “*a mother holding her child and a father or others all around … almost like the Virgin Mary with the Child*” reveal embodied conceptual mappings, in which physical arrangements and gestures represent emotional protection and safety. This theme suggests how metaphors may serve as cognitive anchors, structuring abstract concepts such as support, safety, and care within perceptually grounded schemas.

#### 3.2.2. Instrument (5 Statements)

The second theme, Instrument, includes five statements and captures the functional and goal-oriented aspects of Comfort Care. Participants described Comfort Care as an “*instrument that allows parents […] to embrace the experience, instead of putting it away somewhere […] so that they can go beyond*,” as well as a “*tree*”, “*coat*”, or a “*pillow*” providing protection, support, and guidance. These metaphors reveal a conceptual projection from concrete objects to caregiving functions, indicating that providers cognitively map tangible sources of comfort onto abstract goals such as emotional containment, resilience, and guidance, reflecting embodied and functional schemas.

#### 3.2.3. House (4 Statements)

The final theme, House, comprises three statements and represents Comfort Care as a place of security, cohesion, and life. Descriptions such as “*a big house with a big family*” or “*rich with love*” illustrate how participants cognitively organize abstract notions of compassion, family bonding, and life-affirming care within spatial and familiar schemas. The house metaphor functions as an embodied conceptual frame, connecting spatial, relational, and emotional dimensions and providing a coherent mental representation of the hospice environment and atmosphere. This theme illustrates how metaphors can facilitate the integration of emotional, social, and cognitive aspects of Comfort Care into a unified conceptual framework.

### 3.3. Distribution of Cognitive Domains in Metaphorical Expressions

For each theme, statements were categorized according to the experiential or cognitive domain they primarily invoked (affective, embodied, relational, spatial, or symbolic). The analysis of the 61 statements (death metaphors/analogies *N* = 41; Comfort Care *N* = 20) revealed the distribution of these conceptual frameworks, as summarized in Table 3. The most frequently utilized domain was Symbolic/Spiritual, representing 27.9% of the total sample. This was followed by the Affective/Emotional domain, which accounted for 26.2%. The Embodied/Functional domain represented 16.4% of the expressions, while the Relational domain accounted for 14.8%. Overall, more than half of the metaphorical expressions (54.1%) were concentrated in the Symbolic and Affective categories, making them the predominant frameworks.

## 4. Study 1: Discussion

This first study explored how perinatal hospice care providers use metaphors, analogies, and symbols to conceptualize death and Comfort Care to identify the cognitive and emotional structures underlying these representations. Through open interviews and metaphor elicitation, 61 metaphorical expressions were collected from 14 multidisciplinary team members. Thematic analysis revealed four metaphorical themes related to death (Peace, Nature, Spirituality, and the Unknown) and three related to Comfort Care (Circle, Instrument, and House). Overall, the findings indicate that metaphors function as central cognitive–emotional tools through which professionals organize experience, regulate affect, and construct meaning in neonatal palliative care.

Metaphors related to death predominantly framed dying in non-threatening and meaningful terms, such as peace, natural completion, or transition. This positive framing aligns with previous findings: parents in perinatal hospice contexts often use metaphors of serenity, continuity, and transformation to describe their infants’ deaths (Dahò, 2021; Parravicini et al., 2018). Among the identified themes, Spirituality was the most prevalent. Metaphors of doors, bridges, or butterflies conveyed transcendence and continuity beyond physical death, consistent with evidence that spiritual imagery facilitates meaning-making in end-of-life care (Gathigia et al., 2018; Gregory et al., 2025). From a cognitive perspective, such metaphors may make the concept of death more cognitively accessible while also increasing its emotional salience and making it more tolerable (Gathigia et al., 2018; Guo, 2023). The Peace theme further suggests the activation of schemas that reduce emotional arousal and support professional resilience in the face of repeated exposure to dying (Back et al., 2023). This framing contrasts with practices characterized by aggressive or technologically driven care, where death is often associated with failure, avoidance, or loss of control. As reported by one interviewed: “*In this country, I think we are so aggressive in the treatment that we forget the patient*” (Nurse 3). Accordingly, these biomedical schemas may disrupt meaning-making and emotional regulation, reinforcing distress and defensive practices (Han et al., 2023; Hernández-Gamboa et al., 2024; Rezapour, 2022).

Finally, the theme of the Unknown captured the uncertainty inherent in dying and revealed systematic differences across professional roles. Nurses and physicians, likely due to frequent exposure to physiological decline, tended to employ biologically grounded and immediate metaphors, whereas psychologists, Child Life specialists, and social workers more often used relational, transitional, or phenomenological imagery. This divergence suggests that professional role and clinical proximity to death may shape not only metaphor content but also the underlying cognitive–emotional frameworks through which death is processed and communicated.

Metaphors related to Comfort Care emphasized containment, protection, and relational presence. The Circle theme emerged as a central symbol of cohesion and emotional holding, functioning as a cognitive anchor that may help professionals contain grief and uncertainty while remaining engaged. Its symbolic associations with unity, perfection, and connection (Darabimanesh, 2023; Islam, 2021) likely support the organization of overwhelming experiences into manageable forms. The House cluster similarly highlighted safety, belonging, and relational continuity (Vu, 2020), reflecting the experience of Comfort Care as the creation of a protected environment rather than a set of isolated interventions. In contrast, the Instrument metaphor foregrounded the functional dimension of care, mapping concrete sources of comfort (e.g., a pillow, a coat, a tree) onto abstract caregiving roles and revealing an embodied understanding of support. Across themes, fewer metaphors were generated for Comfort Care than for death. This imbalance may reflect the greater cultural and symbolic elaboration of death, whereas Comfort Care is often implicitly practiced and less explicitly conceptualized. The metaphor-elicitation process itself may therefore have enabled novel reflection, allowing professionals to articulate meanings that are typically taken for granted.

Overall, these findings support the view that metaphors function as cognitive–emotional structures integrating perception and memory (Guo, 2023; Tanaka et al., 2025; Yu et al., 2025). This is reflected in the distribution of metaphorical domains (Table 3), where symbolic and affective schemas (54.1%) highlight their role in processing the weight of perinatal death. These results converge into the proposed Metaphorical Anchor Model, which conceptualizes metaphors as stabilizers for affective overload. Importantly, the systematic variation in metaphor use across roles observed here lays the groundwork for Study 2. In this way, Study 1 provided the theoretical basis to operationalize these anchors as measurable mechanisms in hospice care.

## 5. Study 2: Materials and Methods

### 5.1. Toward a Conceptual Model: Metaphor as a Cognitive Anchor in High-Stress Care

**Figure 1:**
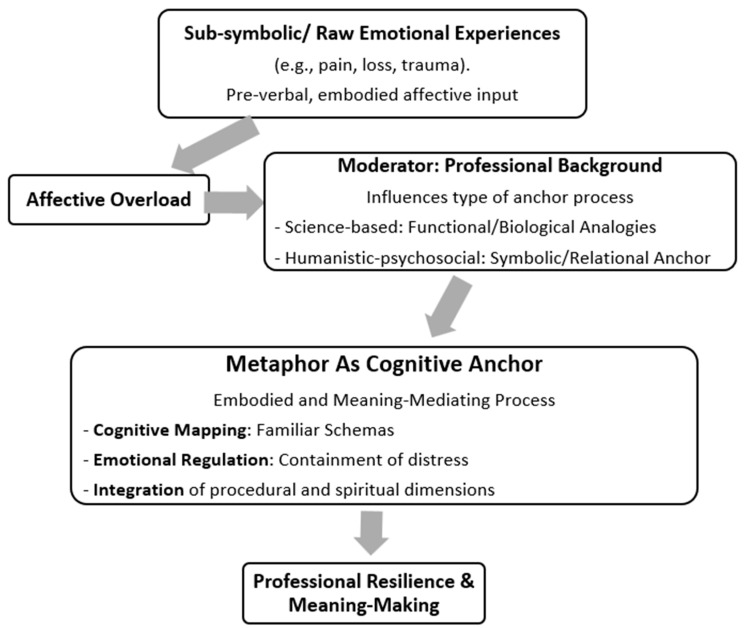
The Metaphorical Anchor Model: A conceptual framework of the cognitive and emotional functions of metaphors in healthcare. The model illustrates the transformation of pre-verbal, sub-symbolic emotional input (e.g., trauma and loss) into structured professional meaning. The process of “Affective Overload” is mediated by the provider’s professional background (the core variable examined in Study 2), which influences the selection of specific anchoring schemas (Functional/Biological vs. Symbolic/Spatial). The central “Metaphor as Cognitive Anchor” box details the mechanisms of cognitive mapping, emotional containment, and the integration of procedural and spiritual dimensions, ultimately supporting professional resilience and identity.

Building on the findings of Study 1, the Metaphor Anchor Model (Figure 1) is proposed as a framework applicable not only to hospice care but to high-intensity healthcare and service professions. In these high-stress contexts, providers are repeatedly exposed to sub-symbolic and raw emotional experiences (e.g., pain, loss, and trauma) that often resist literal language. When this pre-verbal, embodied input is not processed immediately, it leads to affective overload, here conceptualized a state of emotional overload in which the intensity of emotional experience temporarily exceeds the professional’s capacity for cognitive and symbolic representation, consistent with emerging models of emotional saturation in high-demand organizational contexts (Asl et al., 2026; Samoylenko et al., 2026). Within this model, it is hypothesized that professional background functions as a significant moderator of the anchoring process.

While other factors, such as personal beliefs, individual coping strategies, and social support networks, have been widely associated with emotional adjustment and burnout prevention (e.g., Doolittle, 2021), this model focuses on how professional training functions as a cognitive filter. It provides the foundational “safe harbor” from which the professional operates, and when faced with affective overload, it dictates the specific cognitive linguistic strategy employed to transform overwhelming input into structured representations. As seen in Study 1, while a science-based background (physicians, nurses) anchors the providers in functional and biological analogies, a humanistic-psychosocial background (psychologists, social workers) anchors such specialists in symbolic and relational schemas. According to this model, professionals can achieve:

- *Cognitive Mapping*: using familiar schemas to navigate the chaos of the experience;
- *Emotional Regulation*: allowing for the containment of distress;
- *Integration*: linking procedural expertise with the spiritual and existential dimensions of care.

Through this multi-layered cognitive mapping, metaphors enable professionals across different professional service fields to “hold” grief without cognitive overload, often reframing suffering in more acceptable terms. Rather than acting as mere linguistic embellishments, these anchors facilitate *Professional Resilience* and *Meaning-Making*, providing a structured framework that sustains engagement with suffering while supporting the construction of a coherent professional identity.

### 5.2. Data Analysis

To empirically validate the preliminary findings of the Metaphor Anchor Model, Study 2 employs a quantitative design to test whether the use of specific ‘metaphorical anchors’ (Functional vs. Symbolic/Relational) is significantly associated with professional background (Science-based vs. Humanistic). The hypothesis (H1) posits that professional training acts as a critical moderator: while all providers experience affective overload when facing perinatal loss, their specific background dictates the selection of distinct cognitive frameworks for processing these end-of-life experiences. Specifically, it is expected that science-based professionals will rely more heavily on functional analogies to structure the event, whereas humanistic-psychosocial professionals will favor symbolic and relational anchors to contain the emotional impact.

Quantitative data analysis was performed using IBM SPSS Statistics v.29 (IBM Corp, 2023). Given the sample size (*N* = 14) and the categorical nature of the variables, Fisher’s Exact Test was employed to examine the associations between professional roles [Group A: Physicians and Nurses (*N* = 7); Group B: Psychologists, Child Life specialists, and Social workers (*N* = 7)]. The nature of the dataset justifies the use of Fisher’s Exact Test. Unlike the Chi-square test, which relies on large-sample approximations (*N* > 100), Fisher’s test calculates the exact probability of the observed distribution. This approach is particularly recommended when sample sizes are relatively small and when expected frequencies in some cells of the contingency table are low. Furthermore, the test is inherently robust to the numerical imbalance between the two macro-clusters.

Each metaphorical expression was treated as an individual unit of analysis, which could help readers better understand the statistical approach adopted. To facilitate a robust quantitative comparison between professional groups, the six identified cognitive domains (Table 3) were aggregated into two overarching macro-clusters based on their primary functional role within the clinical encounter. The first macro-cluster, named Symbolic & Relational Anchors, combines the Symbolic/Spiritual, Affective, and Relational domains (*N* = 42). These expressions focus on meaning-making, emotional containment, and the interpersonal bond.

The second macro-cluster, labelled Functional & Cognitive Analogies, integrates the Embodied, Spatial, and Cognitive/Unknown domains (*N* = 19) and focuses on the structural, procedural, and descriptive aspects of care. This categorization enables a binary comparison to test the hypothesis that professional background significantly influences the choice between meaning-oriented and process-oriented metaphorical frameworks. The distribution and composition of these macro-clusters are detailed in Table 4.

## 6. Study 2: Results

The results of Fisher’s Exact Test (Table 5) confirm a statistically significant association between professional background and the type of metaphorical anchor employed (*p* = 0.008). This finding provides empirical evidence for the Metaphorical Anchor Model (Figure 1). While the “Science-based” group showed a more distributed use of anchors with a preference for Functional & Cognitive Analogies (54.3%), the “Humanistic” group relied almost exclusively on Symbolic & Relational Anchors (80.8%). The significance level (*p* < 0.01) suggests that the divergence in metaphorical mapping is not accidental but is deeply rooted in the different professional ontologies used to process the affective overload inherent in perinatal hospice care.

## 7. Study 2: Discussion

The quantitative findings of Study 2 provide an initial yet robust empirical validation for the Metaphorical Anchor Model. The significant association between professional background and the type of metaphorical expressions used confirms that professional training acts as a foundational cognitive filter through which perinatal grief is processed and communicated. The most striking result is the overwhelming reliance of humanistic-psychosocial professionals on Symbolic and Relational Anchors. These data suggest that for psychologists, Child Life specialists, and social workers, the “safe harbor” mentioned in the model is constructed through meaning-making and emotional containment. In the face of affective overload, these professionals do not seek structural analogies but rather use metaphors to create a shared symbolic space with the grieving parents. This preference aligns with the ontologies of care that prioritize the “subjective experience” and the “relational bond” over the clinical outcome.

In contrast, science-based professionals (physicians and nurses) displayed a more distributed use of anchors, with a distinct preference for Functional and Cognitive Analogies. This tendency reflects a “technical-scientific” anchoring process: when confronted with the raw emotional input of perinatal loss, these providers use metaphors to “map” the experience back onto structured, procedural, or spatial frameworks. This is not a lack of empathy; rather, it is a specialized form of professional resilience. By translating a sub-symbolic experience into a more “functional” representation, they can maintain the cognitive stability necessary to perform clinical tasks while providing end-of-life care.

Ultimately, the model demonstrates that metaphors are not mere linguistic choices, but key cognitive and meaning-making resources Indeed, In this sense, they may support professionals in “holding” the emotional intensity of perinatal hospice care without succumbing to emotional burnout (Dahò, 2021). The integration of these different “anchors” within a multidisciplinary team ensures that both the procedural needs of the clinical case and the existential needs of the family are addressed.

## 8. Conclusions

The findings of this study indicate that professional role and clinical exposure shape the cognitive and emotional schemas through which death and caregiving are conceptualized. Providers working in perinatal hospice settings tend to employ metaphors emphasizing peace, transition, and continuity, whereas clinicians in emergency or critical care contexts may more often rely on metaphors of urgency, failure, or loss of control (Back et al., 2023; Gregory et al., 2025). Future research should systematically examine differences in metaphor use across professional roles, levels of exposure to death or emergency, and care settings. A central implication of this study concerns the divergence in metaphorical schemas across professional roles. Medical staff predominantly relied on concrete, biological, and “here-and-now” metaphors focused on physiological processes and functional cessation. In contrast, psychosocial professionals more frequently employed relational, transcendent, and spiritually oriented imagery. This ‘semiotic disparity’ reflects broader systemic barriers in healthcare where linguistic or procedural rigidity can inadvertently marginalize vulnerable populations, echoing the exclusion mechanisms and biases often found in clinical protocols and medical healthcare (Albisser Schleger et al., 2011; Dahò et al., 2025; Shepherd et al., 2019). This gap suggests the need to develop metaphorical competence within multidisciplinary teams. Such competence extends beyond the use of figurative language. It may be understood as a clinical skill involving the recognition, translation, and integration of diverse conceptual schemas, thereby supporting a unified and family-centered approach to care (Tay, 2016).

The results also highlight the value of metaphorical thinking as a professional skill that can be intentionally cultivated. Figurative language engages neural systems involved in emotional processing more strongly than literal language, supporting emotional regulation and reflective understanding in high-stress contexts (Citron et al., 2019; Saulsman, 2025; Neefjes, 2023). Training programs or reflective workshops that explicitly foster the use of metaphors may enhance empathy, communication with families, and providers’ capacity to process emotionally intense experiences (Casarett et al., 2010; Kaya et al., 2013; Mathieson et al., 2018; McMullen & Tay, 2023; Saulsman, 2025). Furthermore, metaphor elicitation can serve as a structured tool for professional debriefing (Bowker et al., 2025). Given their familiarity with symbolic expression, Child Life specialists may be particularly well positioned to facilitate interdisciplinary workshops that integrate biomedical information with metaphor-based reflection. Such practices can allow providers to externalize and process abstract or distressing aspects of perinatal loss before they influence clinical judgment or contribute to compassion fatigue. Integrating metaphorical thinking into standard palliative care training may thus provide clinicians with a linguistic “safety net,” helping them organize complex emotional experiences into manageable mental models.

Finally, metaphorical framing influences clinical reasoning and decision-making (Neefjes, 2023; Thibodeau et al., 2017). By externalizing emotionally charged experiences into structured symbolic language, metaphors reduce the immediate influence of raw affect on judgment (Beknazarova et al., 2021; Dahò, 2025; Dahò & Monzani, 2025; Gangemi et al., 2025), supporting more deliberate and balanced clinical decisions (Neefjes, 2023; Thibodeau et al., 2017). Of course, the use of metaphors alone is not sufficient to address all challenges, particularly in situations of high distress, cognitive overload, or burnout. In such cases, structured debriefing sessions, mental health workshops, or the application of targeted techniques, such as CBT-based relaxation strategies (e.g., Chidi et al., 2024; Evans et al., 2023; Gangemi et al., 2019), may be more effective in supporting provider well-being and resilience.

### Strengths and Limitations

A major strength of this study is the inclusion of multidisciplinary participants from three perinatal hospice settings, allowing for a comprehensive exploration of diverse professional perspectives. The integration of metaphorical analysis with cognitive interpretation provided nuanced insight into both linguistic expression and underlying meaning-making processes. Several limitations should, nevertheless, be acknowledged. The small sample size and single-site design limit the generalizability of the findings. Each professional role was represented by a limited number of participants, potentially constraining within-group variability. Moreover, not all participants were accustomed to generating metaphors, which may have restricted the range and complexity of the metaphorical expressions collected. The cognitive interpretations proposed in this study are inferential and based on linguistic data, rather than direct measures of mental processes or emotional states. Finally, the cross-sectional design captures perspectives at a single point in time. It does not allow examination of how metaphor use or cognitive–emotional schemas may evolve across professional experience or repeated loss exposure. Further research is thus needed to explore how metaphors function across different clinical roles, care settings, and cultural backgrounds and to examine their potential impact on communication, decision-making, and emotional processing in palliative care.

## Figures and Tables

**Table 1:** Metaphors, analogies, and symbols related to the concept of death.

Cluster	Quotes
Peace	There is a beautiful grace in a peaceful death (Nurse 1) Death is not suffering (Nurse 2) … is peace (Nurse 2) … is the end of pain (Nurse 3) … is no discomfort (Nurse 3) … is the absence of pain (Nurse 3) … is peaceful (Nurse 4) … is quiet (Nurse 4) … is a place far from tragedies and sadness (Child Life 3) … is peaceful (Social Worker 1) … is a quiet place (Social Worker 2) … is no pain (Physician 1) … is no suffering (Physician 2)
Natural event	Death is a part of life (Nurse 1) … is part of everyone’s life (Nurse 1) … is the completion of this journey (Nurse 2) … is part of a natural course (Nurse 3) … is a great trauma because it is final, it is done, and we cannot undo that (Nurse 4) … is the cessation of the body’s functions (Physician 4)
Spirituality	Death is the door to another part of life (Nurse 2) … is at the beginning of the next part of life (Nurse 3) … is going to a happy place (Nurse 4) … is not an end: it is a part of an infinite circle (Social Worker 3) … is not an end, as people continue to be part of the universe (Social Worker 3) … is flying away truthfully like a butterfly to something positive and beautiful (Child Life 1) … is: spirits and angels who look over families that grieve (Child Life 1) … is an angel (Child Life 2) … is not an end, as people who pass in this life do live on and give us strength (Child Life 3) … refers to another person (God) who takes the initiative with respect to this person who dies (Physician 1) … is another type of hug (Physician 2) … is light (heaven) (Physician 2) … is happiness afterward (Physician 3) … is: going into the arms of Jesus, into heaven, into something good (Physician 4) … is a butterfly and the entire metamorphosis (from being a caterpillar to a butterfly) (Child Life 1) …is a path towards the clouds (Psychologist 1) …is a bridge between here and the hereafter (Psychologist 2)
Unknown	Death is a big question mark (Physician 1) … is a mystery (Physician 2) … is something unknown (Nurse 4) …is something mysterious… (Psychologist 1) …is a road one travels, leaving things behind, without knowing what lies ahead (Psychologist 2)

**Table 2:** Metaphors, analogies, and symbols related to Comfort Care or Perinatal Hospice practice.

Cluster	Quotes
Circle/Union	In the Comfort Care unit, it is like we hold each other’s hands (Child Life 1). Comfort Care is a circle because the circle symbolizes the family unit and the medical team (Child Life 2). … is the circle of birth and death at the same time (Child Life 2); … is a mother holding her child and a father or others all around, almost like the Virgin Mary with the child, with a look of peace and joy (Nurse 1); … is like a mother hugging her child (Physician 1); … is a hug proportioned to the measures of what you embrace (Physician 2); … is a mother’s arms because in a mother’s arms is where a baby feels safe (Physician 3); … is two hands holding each other (Psychologist 1); … is walking arm in arm, until the final goodbye (Psychologist 1); … is a caress (Psychologist 2); … is an embrace (Psychologist 2);
Instrument	Comfort Care is like a beautiful wild tree […] with all the information, comfort, and support. […] A tree with many green and big leaves and very strong roots and branches (Child Life 2) … is like a warm coat that makes families feel warm and protected (Child Life 3) … is an instrument that makes a lot of families understand that their experience is part of their path (Social Worker 1) … is an instrument that allows parents […] to embrace the experience, instead of putting it away somewhere […] so that they can go beyond (Social Worker 3) …is a pillow comfortable and of comfort for the dying baby and the family around (Nurse 4)
House	Comfort Care is a place of compassion, painless, a time of peace, family cohesiveness, and life (Nurse 2) …is like a big house with a big family (Nurse 3) … is a house rich in love, peace, and families (Social Worker 1) … is a place of life, even if life here is short (Social Worker 2)

**Table 3:** Frequency of Conceptual Domains Across Metaphorical Themes.

Domain	N of Statements	Percentage of Total (61)
Symbolic/Spiritual	17	27.9%
Affective/Emotional	16	26.2%
Embodied/Functional	10	16.4%
Relational	9	14.8%
Cognitive/Unknown	5	8.2%
Spatial	4	6.5%

**Table 4:** Macro-clusters of metaphors and analogies.

Macro-Cluster	Domains	Total (*N*)	Percentage
Symbolic & Relational Anchors	Symbolic (17) + Affective (16) + Relational (9)	42	68.85%
Functional & Cognitive Analogies	Embodied (10) + Spatial (4) + Cognitive/Unknown (5)	19	31.15%

**Table 5:** Fisher’s Tests for Professional Background and Metaphorical Clusters (*N* = 61).

Test Type	Value	Asymptotic Significance (2-Sided)	Exact Sig. (2-Sided)	Exact Sig. (1-Sided)
Pearson Chi-Square	7.682	0.006	-	-
Continuity Correction	6.283	0.012	-	-
Likelihood Ratio	8.053	0.005	-	-
Fisher’s Exact Test	-	-	0.008	0.005
Linear-by-Linear Association	7.556	0.006	-	-
N of Valid Cases	61	-	-	-

## Data Availability

Data are available upon reasonable request from the corresponding author.
